# A Comprehensive Assessment to Enable Recovery of the Homeless: The HOP-TR Study

**DOI:** 10.3389/fpubh.2021.661517

**Published:** 2021-07-09

**Authors:** Coline Van Everdingen, Peter Bob Peerenboom, Koos Van Der Velden, Philippe A. E. G. Delespaul

**Affiliations:** ^1^Department of Psychiatry and Neuropsychology, Maastricht University, Maastricht, Netherlands; ^2^Tangram Health Care Consultancy, Doetinchem, Netherlands; ^3^Department of Primary and Community Care, Radboud University Medical Centre, Nijmegen, Netherlands; ^4^Department of Adult Psychiatry, Mondriaan Mental Health Trust, Heerlen, Netherlands

**Keywords:** homelessness, severe mental illness, transdiagnostic mental health strategy, InterRAI Community Mental Health questionnaire, human rights, recovery, public health policy, healthcare ecosystem approach

## Abstract

**Background:** Homelessness is an increasing problem in Western European countries. In the Netherlands, policy reforms and austerity measures induced an urgent need for management information on local homeless citizens. Municipal authorities initiated cross-sectional reviews of Homeless Service (HS) users. The resulting Homeless People Treatment and Recovery (HOP-TR) study developed a health and needs assessment strategy over different domains to comprehensively assess individuals and care networks with the perspective on recovery.

**Methods:** Dutch HS users were selected using a naturalistic meta-snowball sampling. Semi-structured interviews provided the primary data source. The interview content was partly derived from the InterRAI Community Mental Health questionnaire and the “Homelessness Supplement.” Using the raw interview data, algorithmic summary scores were computed and integrating clinical parameters assessed. The data describe health and needs in a rights-based, recovery-oriented frame of reference. The mental health approach is transdiagnostic. The positive health framework is used for structuring health and needs aspects in relation to the symptomatic (physical and mental health), social (daily living, social participation), and personal (quality of life, meaning) dimensions of recovery.

**Results:** Recruitment (between 2015 and 2017) resulted in a saturated sample of 436 HS users in 16 facilities and seven cities. Most participants were long-term or intermittently homeless. The sample characteristics reveal the multi domain character of needs and the relevance of a broad, comprehensive approach. Local authorities used the reports to reflect and discuss needs, care provision, access, and network cooperation. These dialogs incited to improve the quality of care at various ecosystem levels.

**Discussion:** This paper describes new recruitment strategies and data collections of comprehensive data domains, to improve our knowledge in the field of homelessness. Traditional epidemiological literature on homelessness is often domain specific and relies on administrative sources. The HOP-TR study uses an analytical epidemiological approach. It shifts the assessment focus from problem-centered marginalization processes toward a comprehensive, three-dimensional recovery-oriented vision of health. Different perspectives are integrated to explore the interaction of homeless people with care networks.

## Introduction

Homelessness is a wicked problem, a loaded phenomenon, inconspicuously triggering exclusion mechanisms associated with social and moral injustice. Homeless people face substantial disadvantages in health and social functioning. Not having a home has a negative impact on their autonomy and quality of life. It is associated with high morbidity and mortality rates (1–4).

Chances to become homeless are unequally distributed. There is consensus, that homelessness is the result of a complex interplay of individual vulnerabilities and interpersonal, structural, and systemic factors. Diverse multilayered and interrelated risk factors pave the way to marginalization (5–8).

Since the mid-nineties, homelessness typologies were used to analyze inequalities and disclose underlying cracks in welfare systems (9–11). Regarding typologies, Kuhn & Culhane's cluster analyses in the public shelters of New York City and Philadelphia yielded innovative insights (9). They observed that episodic and chronic homeless people had a substantially higher prevalence of Severe Mental Illness (SMI), substance use, and medical problems. The chronic homeless cluster (10%) consumed half of all shelter days. In advanced welfare systems, with an elaborated, siloed spectrum of services, people with chronic and complex care needs are more at risk to be underserved and stay homeless (10, 12). Mental illness, substance abuse, and physical illness often generate complex care needs resulting in homelessness (13–18). Siloed care systems do not serve them well.

During the nineties, in the USA, homeless people with SMI and addiction were targets of integrated case management models such as Assertive Community Treatment (ACT) or Intensive Case Management (ICM) (19). Both models provide comprehensive ambulant care in a staff – care user ratio of about 1 to 10. Both include intensive care, outreach, treatment, and care coordination, but only ACT teams work with a shared caseload. Housing First, including ACT or ICM, proved being a successful intervention for mentally ill homeless individuals (20, 21). Still, specific needs related care is organized in siloed services, which are insufficiently capable to serve complex patterns of problems and needs of marginalized groups (22, 23). Even in Denmark, where one of Europe's largest Housing First Programs is embedded in a national homelessness strategy, only one out of twenty homeless people is served.

Despite our knowledge on its etiology and evidence based strategies to end it, homelessness remains a pervasive, increasing problem in Western European countries (24). The epidemiological homelessness literature shows several gaps. First, the population is difficult to access. Second, for various reasons, health and needs data on homeless citizens is fragmented and incomplete. Further, the information gap significantly relates to the question how services are profiled and interact. How to create room for improvement? How to shape better conditions – in addition to housing – for recovery in marginalized populations with interdependent needs? How to mirror and foster adaptive capacities at the levels of individuals and networks?

In the Netherlands, a need of comprehensive and accurate information on homeless inhabitants incited new research. For service planning goals, Homeless Service (HS) organizations and municipalities commissioned local reviews to obtain management data on health and needs of HS users. An integrating assessment approach was developed to collect health data in order to open new perspectives to this underserved part of the Dutch inhabitants. The commissioning organizations gave permission for scientific use of the management data in the Homeless People Treatment and Recovery (HOP-TR) study. The research ethics committee of the Radboud University Nijmegen Medical Centre certified that the research does not fall within the remit of the Medical Research Involving Human Subjects Act (file number 2018-4463).

Other papers will present the profile of the HOP-TR sample and the health patterns of Dutch HS users (25). This methods paper describes the comprehensive recruitment and assessment strategy of the HOP-TR study. It explains which elements were added to existing instruments, to enable a shift from marginalization to recovery. It documents, for instance, how collected data were used to assess mental health in a transdiagnostic approach. The Supplementary Materials provide a complete overview of all variables, to enable replication.

## Contextual Factors Demanding for New Research

### Revision of Health Concepts

In the paradigms underlying health and social security systems, biomedical and categorical health approaches are still dominant. Evidence based practices founded on these models may fit well to “clear-cut” disease, but bring disadvantages if health problems are having a more complicated or long-standing character. The overemphasis on medical treatment of symptomatic health aspects, tends to ignore the relevance of social and personal health dimensions. Consequently, the human need of personal autonomy is easily neglected. Fortunately, these mechanisms and concomitant care gaps have been addressed (26–28).

Pointing to mental health as well, more and more authors discern these disadvantages, and develop alternative, integrated health strategies (28–31). In 2011, Huber et al. proposed an alternative, more dynamic perspective on health. The revised concept considers “health as the ability to adapt and self-manage, in the face of social, physical and emotional challenges” (29). Its evaluation among different stakeholder groups resulted in the positive health framework. The framework counts six health domains: physical health, mental health, daily functioning, social participation, quality of life, and meaning (30).

In 2015, Manwell et al. (31) reported on an international, interdisciplinary, exploratory survey induced by the lack of a shared vision on mental health. Part of the experts participating in this dialog were people with lived experience. The dialog resulted in the proposal of a transdomain model of health, based on Huber's positive health definition and the WHO's definition of mental health (32, 33). In this transdomain model, the overlap of the three (mental, physical, and social) health domains are used to define autonomy, sense of “us,” control for navigating social spaces, and agency. Recognizing the significance of human rights, for all three domains a human rights standard of functioning and adaptation was proposed.

In 2019, Van Os et al. (28) provided a complete, clear-cut analysis of the disadvantages of the evidence-based group-level symptom-reduction model, that dominates current practice in mental health. Regarding the character of mental health problems, the authors plead that clinical practice is better served by a patient-centered trans-syndromal framework, allowing to flexibly mix-up categorical, dimensional and network approaches. According to the authors, 10 to 15 transsyndromes, going across and beyond diagnoses, are sufficient to cover daily clinical practice. Finally, the consequences of a transsyndromal mental health approach to the organization and design of mental health services are discussed.

In 2020, Fusar-Poli et al. (34, 35) critically reviewed the literature about self-defined transdiagnostic approaches. The authors observed that transdiagnostic approaches aim to improve the classification and treatment of mental health problems by cutting across and going beyond categorical ICD/DSM diagnoses. They formulated the “Mansell criteria” to assess the transdiagnostic character of each approach. The review revealed the unstandardized use of the term “transdiagnostic.” Most studies tested transdiagnostic features across, only few studies beyond diagnoses. The authors conclude that current clinical empirical evidence is insufficient to endorse a paradigm shift.

In his editorial letter responding to this review, Mansell uses the literature on the general psychopathology factor “p” to illustrate that literature relevant to the transdiagnostic approach goes beyond the word “transdiagnostic” (36). Transdiagnostic approaches aim to identify, utilize, and test a general theory of psychopathology. Besides symptom relief, Mansell names efficiency, cost-effectiveness, accessibility, and patient-reported distress reduction as examples of valuable outcome measures to evaluate the impact of transdiagnostic interventions. Though in treatment evaluations randomized controlled trials are considered the gold standard, Mansell notes that building and evaluating theories in a process approach still is the most robust way to test them. Finally, he concludes that treatment research needs to consider the perspectives of different stakeholders, while process research needs to be theory driven.

Homeless people are also affected by the dominant paradigms and current evidence based standards. The epidemiological literature on homelessness is extensive, but often domain specific and relying on administrative sources. It mainly focuses on accumulations of risk factors and structural factors resulting into marginalization, but less frequently highlights personal strengths or human need for autonomy and freedom of choice (5–8, 37). Recent literature on effective strategies to end homelessness, such as the At Home Chez Soi Program in Canada, is very rich (20, 38–41). Program development, its constituents, and its effects on various recovery aspects are well-documented. Unsurprisingly, perspectives of care users and qualitative aspects are included in this literature, since Mad Studies emerged at Toronto (41–43). Still, the homelessness literature mainly highlights positive results, while only few papers evaluate why proven effective interventions or programs do not always work (44, 45).

It is taken for granted that SMI is an important risk factor that increases marginalization and can result in homelessness (13–18). The overall population prevalence of mental illness is 1 out of 4 (24%). In the American literature, SMI is applied to people in need of care (6%). However, in The Netherlands the predicament “severe” is reserved to individuals in need of integrated care (combining specialized psychiatric care and welfare strategies). In 2013, Delespaul and a Dutch Consensus Group revised the EPA^1^ definition to validate criteria for individuals in need of comprehensive care solutions and allow access to upgrade service delivery in the context of rising costs of care (46). People are part of the SMI/EPA population if (1) they have pervasive mental illness symptoms giving rise to impairments in personal functioning and societal participation; (2) when interrelated symptoms and disabilities trigger each other (circularity); and (3) when integrated and coordinated professional care is required to realize change. In 2013, the estimated prevalence of SMI/EPA in the adult Dutch population according to this revised definition is 1.7%. This means about 177.500 adults with SMI/EPA in the Netherlands in 2020.

This Dutch SMI/EPA definition acknowledges the fluctuations in the symptomatic, social, and personal impact of pervasive mental suffering. It offers a broad, integrated perspective on the three domains of recovery: symptom relief, social participation, and personal recovery (47–50). The importance of these three domains for recovery in SMI is generally admitted.

New assessment instruments, such as the Social Outcomes Index (SIX) and the Functional Recovery scale (FR-scale), were developed to include social and personal domains in the routine outcome monitoring of SMI (51, 52). Research with the FR-scale demonstrated its practical usefulness and validity to measure changes in the social participation domain. This is relevant for policy evaluation and practices and implemented in regular evaluation of Flexible Assertive Community Treatment (F-ACT) teams and organizations for sheltered and supported living (53).

### Siloed Care

The need of integrating multidisciplinary care strategies for severely mentally ill people with interdependent care needs is generally admitted. Many authors demonstrated the relevance of interpersonal relationships and care coordination to meet care needs in this group (12, 22, 54–58). Still, poor implementation of this knowledge into practice results in fragmented, siloed, care. In countries with low poverty rates and extensive welfare systems, homelessness mainly affects people with interdependent needs (10, 12, 22). In such countries, a substantial part of the homeless people is intermittently or long-term homeless. Unfortunately, literature on the interaction between service users and systems is scarce. Though, the coverage rates of proven effective interventions are low, there is still little knowledge why people fall out of trajectories or what they need in addition to housing and case management. Knowledge on the perspective of homeless people themselves on health, priorities and care perceptions is scarce. Similarly, only few studies included both perspectives of HS users and professionals to collect data on their mutual interaction.

Nicaise et al. (12) applied social network analysis to evaluate the collaboration of mental health organizations and social services in deprived areas of Brussels and London. They demonstrated that collaboration within services was much higher than between services. In fact, their study also revealed the need of supportive strategies to foster collaboration in care networks serving marginalized populations with complex needs.

Recently, Rosen, Gill, and Salvador-Carulla explicitly addressed the failure of mental healthcare systems to meet complex multisectoral care needs, such as in homeless populations (59). They revised the literature on critical elements at individual and regional/national level. They argued to reframe community healthcare in a healthcare ecosystem approach, targeted at balanced care (60) and rooted on the keystones of community mental health (person-centeredness, recovery, human rights, challenging stigma and discrimination). Such ecosystem approaches can enlarge adaptive capabilities of care networks for marginalized populations, if “one size fits all” or “one chain of care” does not fit. They intend to offer comprehensive frameworks to translate assessment data, models, and scenarios into useful policy and decision support information (61, 62). The frameworks coherently consider information at different levels in the Mental Healthcare Matrix model (63), e.g., at nano- (individual), micro- (service of organization), meso- (local) and macro- (regional of national) level. Various types of information serve as input to foster dialog at different levels, which supports the conjoint process of taking responsibility and shaping care. Ideally, “hard” factual data is combined with “soft” information on personal values. Obviously, people being served should be included as natural partners at all ecosystem levels. Currently, ecosystem approaches are implemented in mental health care in various countries and at different scales, such as in Australia and Bizkaia (61, 64).

### Policy Reforms

In the Netherlands, people without an address fall off the governmental radar. Since they cannot be identified in the Basic Registration of Persons, they are often excluded from monitoring, therefore underrepresented in social surveys. Homeless services are embedded within the social welfare domain. Medical records are not available; intakes are done by professionals without knowledge or skills to systematically collect and interpret health information. In addition, insurance problems and overriding priorities interfere with access and engagement with care. This results in a lack of comprehensive health information.

Official homelessness estimates indicate that the number of homeless people in 2018 had more than doubled since 2009 (65). Several factors in Dutch society induced local authorities to monitor the health of homeless citizens. Since 2014^2^ more signals of confused and homeless people in the streets were reported. This occurs in a country with high mental health care consumption, that recently started a national plan to reduce hospital beds and develop more ambulatory care. The centralized Dutch housing policy failed to end housing shortages and put fundamental human rights at stake (66). From 2015 on, austerity measures were implemented to safeguard the Dutch social security system. The responsibilities of municipalities for disadvantaged and disabled citizens expanded, assuming that local parties are better placed to coordinate care. Consequently, local authorities were burdened with increased responsibilities and diminished funding. At the same time, in December 2014 the national monitoring system (National Strategy Plan for Social Relief) was stopped (67, 68). From 2015 on, policy reforms, austerity measures, and media attention to confused people in the streets were setting the scene.

## Study Design

### First Local Review

In March 2015 the Salvation Army, the largest homeless service (HS) provider in the Netherlands, commissioned an independent researcher (CvE) with a professional background as a medical doctor to conduct a health review in one of its night shelters. A participative research approach with semi-structured interviews was used to collect health data in a sample of HS users. The review results were shared with regional stakeholders and pushed the national debate. The review was repeated in another region. In December, the Salvation Army presented the review report to the minister of health. This report, providing detailed information on HS users' health and needs, illustrated the interdependent needs, and the pervasive character of homelessness of HS users in the reviewed services. It stimulated discussion on quality and efficacy of care in those services and elsewhere.

From 2015 on, the context of policy reforms with austerity measures induced local authorities to commission local reviews among homeless services users. Therefore, the review strategy was replicated by local authorities in other settings, to fulfill their information needs.

### Objectives and Design

The first review was the starting point of the Homeless People Treatment and Recovery (HOP-TR) study as well. The original researcher liaised with scholars from public (KvdV and PBP) and mental health (PhD). A multistage cross-sectional design was used to collect health and needs data in different shelters and homeless services. In all settings, semistructured interviews were used to collect data in a sample of the HS users. All interviews were conducted by an independent researcher with a professional background as a medical doctor (first author: CvE). From the first review on, a comprehensive assessment strategy was used to collect relevant health data over different areas of life.

The HOP-TR study aims to identify which conditions can promote sustainable recovery in marginalized populations with interdependent needs. Goals are to better deal with interdependent problems both at the level of individuals and care networks. Therefore, the HOP-TR study has three objectives:

1. To describe the health of Dutch Homeless Service users, including different health aspects and comorbidity;
2. To analyze patterns of marginalization and disclose care gaps in interactions of service users and systems;
3. To identify conditions to promote recovery in marginalized populations with interdependent needs.

During the study, the assessment strategy was further developed to optimize the collection and use of data related to the study objectives. The reasons behind and content of the additions will be explained.

## Recruitment Strategy

### Setting Recruitment

Recruitment followed a dual snowball sampling process (69, 70): sampling of settings (meta-snowball) and sampling of individuals within settings (individual snowball). After presenting the first report to the minister of health, local authorities spontaneously required their own reviews. The on-going public debate at local, regional, and national levels incited local authorities to fulfill their information needs. It kept the meta-snowball rolling: additional settings were not selected by plan, but naturally added on request. Researchers monitored the regional spreading and facility type, to ascertain the diversity of the sample. Included facilities represent the various HS facility types in the Netherlands: night, daytime, and crisis shelters, and protected living services for homeless people. Only settings for adult HS users were included. Settings were added until topic saturation was reached, as long as reports generated new insights in health patterns. The meta-snowball process started in March 2015 and ended in November 2017.

### Participant Recruitment

In each center, the study was introduced with posters and leaflets. To lower the contact threshold, the researcher participated in program activities. For example, she used the afternoon opening session or the evening meal to introduce herself and provide information about the study. Subjects were asked whether they were willing to participate. Original subjects were the seeds of the subject-level snowball. They introduced the researcher to other homeless people in their network. The resulting subsample comprised original subjects and persons recruited through nomination. Its representativeness was checked with the facility staff. When specific subject profiles were missing, staff introduced the researcher to a typical user. The naming procedure ascertained going beyond the most easy to access subjects, to obtain a representative diversity of the subject sample. This strategy ascertained inclusion of a diversified group of HS users in each setting. To optimally cover population diversity, recruitment aimed to interview most HS users present in the facility at the interview days.

At the start of each interview, the anonymized data storage and reporting were explained. When subjects were insufficiently fluent in Dutch, interviews were conducted in English, German, or French. In 2% of the interviews, an interpreter was arranged. Each interview lasted between 1 and 2.5 h. The participants provided informed consent and received a small (5–7.50 €) voucher for completing the interview. Many participants appreciated the researcher's true personal interest and experienced the interview as empowering and hopeful. Some HS users had to be invited repeatedly. HS users who declined participation, motivated their decision by having other priorities, or language problems.

## Assessment Strategy

### Fundamentals of the Assessment Approach

From the first review on, the results of the comprehensive interview approach revealed unmet needs in many life domains. Despite accumulations of health problems and vicissitudes, many HS users were underserved. Also, feelings of disappointment and indignation about the quality of services, or the way people had been treated, were frequently uttered. In their perspectives, services and/or society had let them down. These observations asked for a more careful examination. In the course the study, the basic assessment strategy was extended. A comprehensive assessment approach was developed founded on six fundamentals:

- it takes the HS users' perspective as the primary reference;
- it uses a transdiagnostic mental health strategy;
- it integrates the HS user's and professional perspectives to gain insight into interactions of homeless people and care networks;
- it uses the positive health framework to structure health data;
- it shifts the assessment target from marginalization trajectories to recovery processes;
- it applies a rights-based approach to assess needs.

### Data Collection, Retrieval, and Processing

The HOP-TR data were collected in different settings and combined into one file. In each setting, different information sources were documented and integrated. The interviews provided the basic data. This was supplemented, when available, with information provided by the commissioning organizations: information about referrals, intakes, care histories and current care. Medical records were only available in the protected living facility. Data were entered in digital Case Report Forms (CRFs). Raw interview data were recoded to disclose relevant meta-concepts. For that purpose, algorithmic summary scales were used. Additionally, several custom codes were defined to better meet the objectives of the HOP-TR study. They were considered necessary to assess modern state-of-the-art health and care needs in a rights-based, recovery-oriented perspective.

Table 1 presents the core elements of the HOP-TR assessment approach and the main topics of the collected data. The three compounds successively display the assessments during the basic interviews, the algorithmic summary scales, and the clinical integrating assessments. The following subsections briefly convey focus, origin, and operationalization of the various parts in this approach.

**Table 1:** HOP-TR assessment approach.

	**Name**	**Main topics**	**Items**
Basic Interviews	Open questions	Nuclear family; life course; living situation (homelessness, migration); social context; education; work history; daily activities; personal goals; vulnerabilities; strengths	12
		Care history, care perceptions	
	Community Mental Health questionnaire	Presence and degree of mental, physical, and social health conditions over different areas of life	346
	Homelessness Supplement	Homelessness history, migration background, social network, education, working experience, income	**71**
	Montreal Cognitive Assessment	Cognitive impairments	30
	Screener for Intelligence and Learning Disabilities	Intellectual impairments, literacy	14
	Quality of Life questionnaire Quality of Care questionnaire	Life, living situation, social relations, physical health, mental health;	7
		Quality of care, confidence in case manager	
	Camberwell Assessment of Need	Presence & fulfillment mental, physical, social needs in different life areas	29
	Functional Recovery scale	FR daily living & self-care, FR work study household, FR social contacts	3
Algorithmic Summary Scales	CMH CAPs & scales	Positive symptoms, depression, mania, traumatic life-events, sleep disturbance, cognitive performance, substance use, smoking, exercise, aggressive behavior, harm to others, self-harm, self-care, interpersonal conflicts, informal support, social relationships, criminal activities, personal finance, education & employment, weight management, ADL, pain	22
	Social Outcomes Index	Employment, Accomodation, Family, Friends	4
	European Typology of Homelessness and Housing Exclusion	Current homelessness	2
		Previous homelessness	
Clinical Integrating Assessments	Chronic Physical Health Problems	Cardiovascular; gastrointestinal; infectious; musculoskeletal; neurological; respiratory; endocrine; malignancy; weight; visual; auditory	**14**
		Physical Health Problems (sum score)	
	Transdiagnostic mental health features	Addiction, anxiety, trauma, depression, psychosis, agitation or aggression, problematic personality, intellectual impairments, neurocognitive impairments, identity, gender, somatization	**4**
		Mental Health Problems (sum score)	
		Mental Illness	
	Concurrent Health Problems	Mental illness, addiction, intellectual impairments, chronic physical health problems	
	Mental Health Related Care-Needs	Presence & character of care needs related to (severe) mental illness	**10**
	Future Living Status	Rights-based assessment of optimal residence after leaving homeless services	
	Care-Needs appraisal	Independent, rights-based professional care-needs appraisal informed by current needs	

*The right column shows the number of items to assess. The bold figures relate to newly defined items in the HOP-TR approach*.

### Basic Interviews

Regarding to the basic interviews in Table 1, each interview started with *open questions* to explore and collect data on the personal biography and the personal perceptions of care and needs. Five questions made up the thread in the narrative interview part: Where are you from? What has happened to you? What are your vulnerabilities, what are your strengths? What are your desires regarding your life? What do you need? The questions originate in analyses on patient-centeredness and successful therapeutic relationships (71–73). The narrative information in response to these questions covers the main topics summarized in Table 1.

In addition, the Dutch version of the *Community Mental Health questionnaire* (CMH, version 9.2) was used to collect health data over different areas of life (74). The CMH collects both data in participants' and professional perspectives. The CMH is developed for use in community mental health agencies. It belongs to the interRAI Suite of Mental Health assessments instruments, designed for integrated assessments and patient-centered care planning in vulnerable populations with complex needs. An overview article extensively documents focus, topics, psychometric properties, and current applications of these instruments (75).

Further, the *Homelessness Supplement* (HSup) was added to systematically collect information homelessness history, social, and personal resources. The HSup is largely derived from the Dutch Strategy Plan for Social Relief (68). New codes such as work status were added, which merges lifetime working experience and current work into one single code. Supplementary Table 2 contains a complete description of all HSup codes.

On indication, the *Montreal Cognitive Assessment* (MoCA) and the *Screener for Intelligence and Learning Disabilities* (SCIL) were used. The MoCA is designed to screen mild and moderate cognitive impairments (76, 77). The SCIL assesses mild intellectual disabilities and borderline intellectual impairments (78, 79). Simultaneously, it was used as a literacy screener to supplement interview data on school career.

The *Quality of Life/Quality of Care questionnaire* is a brief self-assessment scale to measure perceptions on Quality of Life (QoL) and Care (QoC) (80, 81). It uses a Likert scale, ranging from 1 (not at all), 4 (fair) to 7 (very).

The *Camberwell Assessment of Need* (CAN) was added to systematically register met, unmet and overmet needs. The CAN was scored in accordance with the manual of the Cumulative Needs for Care Monitor (81, 82). Scores integrate the participant's and the researcher's professional point of view. Each need domain was coded in accordance with the latest version of the CAN (83), which uses a nominal scale to assess presence and fulfillment of care needs: N (No need) assigns the absence of care need because independent functioning in that domain; M (Met needs) indicates that observed care needs are met; U (Unmet needs) points to an incomplete or absent coverage of care needs; finally, O (Overmet needs) is a signal of potentially overmet needs.

The *Functional Recovery (FR-) scale* is a clinical assessment scale to evaluate social functioning and functional recovery of people living with SMI over the past 6 months (51). Assessments are made in reference to expected functioning of people of the same age and social context without any limitations due to (mental) health problems. The three scale domains are assessed with the number codes of the original CAN (0: no problem; 1: covered or light problem; 2: severe problem).

### Algorithmic Summary Scales

Concerning to the algorithmic summary scales in Table 1, the *CMH CAPs and scales* are meaningful risk indicators and quick needs assessments, relevant to daily functioning. The CMH contains a set of decision rules yielding the CAPs and scales conform with in the CMH User's Manual (60, 61). The *Social Outcomes Index (SIX)* measures social and personal aspects relevant to recovery (52). *European Typology of Homelessness and Housing Exclusion* (ETHOS) was used to assess the homelessness typology and prior homelessness. FEANTSA, the European NGO fighting against homelessness, developed this typology as a cross-national reference framework to enable discussion and engagement in this field (84). It applies to homeless people, whether they are roofless, houseless, or living in insecure or inadequate homes.

In this study, the CMH algorithms were applied to compute the CAPs and scales directly after digital data entrance. Raw interview data were used to compute the results on the SIX.

Further, raw homelessness data were reviewed to assess previous and current homelessness.

### Clinical Integrating Assessments

All clinical integrating assessments were newly defined for the HOP-TR approach. In contrast with the algorithmic summary scales, these assessments rest on a clinical appraisal of collected data.

*Physical Health Problems* records chronic health problems, resulting into persistent impairments in daily functioning and/or structural care needs, which are relatively common in homeless populations. When concomitant diseases in one organ system were observed, only the most severe one was coded. For example, if signs were present of esophagitis and liver cirrhosis, the code of liver cirrhosis was used. In addition to the scores of separate organ systems, the sum scores were computed to count the number of Physical Health Problems in all subjects.

*Transdiagnostic Mental Health Features* reflect the presence of major complaints and behaviors, which qualify current mental health (28, 85). In the HOP-TR approach, the transdiagnostic features are prevalences over the past 3 months. They identify conditions relevant to current functioning as well as to the course of participant's life. In contrast to DSM-5 group diagnoses, implicit symptoms are not discarded. For instance, fear/anxiety is coded irrespective of its occurrence as part of a psychotic disorder. Intellectual disabilities and neurocognitive impairments were only scored if the suspicion is supported by additional symptoms in life history, school career, clinical observation, and/or screener results. Attachment problems were common and are coded as part of impulse disorders or personality problems. Identity problems are only coded if severe attachment problems determined the participant's life course.

The number of transdiagnostic features were used to compute the burden of Mental Health Problems in all subjects. Instead, *Mental Illness* counts the presence of any transdiagnostic features, excluding Addiction, or Intellectual Impairments.

*Concurrent Health Problems*, uses the dichotomized scores of Physical Health Problems, Mental Illness, Addiction, and Intellectual Impairments to count how many of the four health domains are affected.

**Figure 1:**
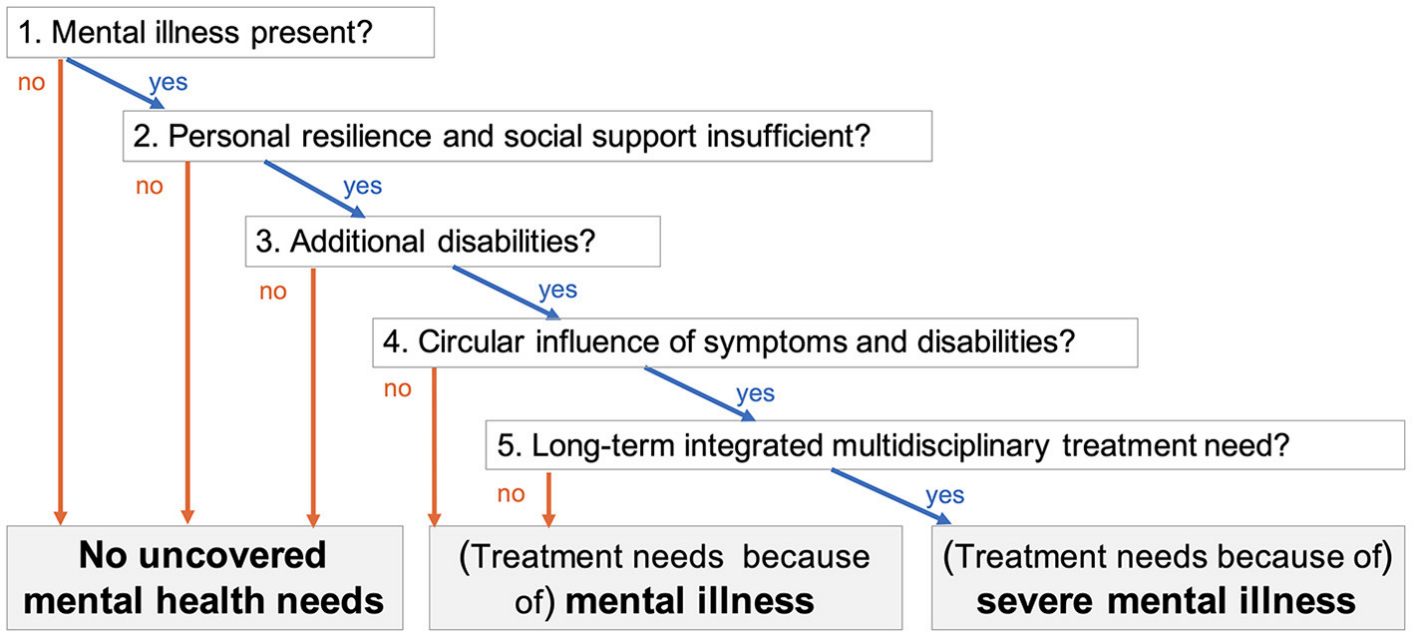
Decision tree to assess Mental Health Related Care Needs.

The decision tree in Figure 1 is based on the Dutch consensus definition of Severe Mental Illness EPA (46). It uses longitudinal information on health patterns to differentiate between absent needs, mental illness, and severe mental illness. It offers a pragmatic clinical assessment tool to assess *Mental Health Related Care Needs*.

*Future Living Status* is a rights-based custom code to assess the optimal residence after leaving homeless services. The participant's needs were leading, instead of the current organization of care and services. The code integrates the participant's and the researcher's professional point of view, like scoring instructions of the CAN in the Cumulative Needs for Care Monitor. The code is biased toward the ambulatory care, since even very intensive treatment and support can be offered in an ambulant setting. The code value “independent living” reflects this.

The code *professional Care-Needs Appraisal*, reflects the results of an independent professional appraisal informed by actual needs. Like Future Living Status, assessment scores are not restricted to supplied services in the current setting. Again, human rights, that foster freedom of choice, and optimal personal autonomy, are leading.

Together, the interview assessments and the recodings in the HOP-TR approach offer a framework to assess HS users' health and needs and to analyze service user – system interactions in a rights-based, recovery-oriented approach. It uses quantitative data for assessing various health and needs aspects, while qualitative data provide additional insight into the meaning of the data. The right column of Table 1 displays the numbers of assessed items; the bold figures distinguish newly defined items from established codes. The bold figures show that the HSup contains 71 items. The clinical integrating assessments comprise 38 health and needs assessments. Supplementary Table 1 depicts the subjects, main topics, and the various sources of data in the HOP-TR database. Supplementary Table 2 presents a complete overview of all Homelessness Supplement codes and all clinical integrating assessments.

## Results

### Recruitment Results

Table 2 presents the results of the naturalistic snowball sampling strategy at the level of settings and subjects. It shows that data was collected in 16 facilities in seven cities, resulting in a total of 436 interviews. The sample consists of adults who had the right to use HS. In general, the possession of a valid residence permit was a prerequisite to access the HS. Since the daytime shelter was open to all inhabitants, eight participants without a valid residence permit were included in the study.

**Table 2:** Setting recruitment.

**Subsample**	**Year**	**Period**	**Commissioning organization**	**City**	**Facilities**	**Size (% of caseload)**
1a	2015	March–April	HS provider	Heerlen	1 night shelter	57 (72%)
1b		September		Dordrecht	1 night shelter	51 (75%)
2	2016	March	Municipality	Landgraaf	1 crisis shelter	35 (70%)
3a	2016	May	Municipality	Utrecht	2 crisis shelters	31 (72%)
3b		June			2 night shelters	61 (59%)
4	2016	October	HS provider	Eindhoven	1 daytime shelter	40 (open group)
5	2017	April–May	Municipality	The Hague	1 protected living facility	28 (76%)
6a	2017	May–June	2 HS providers	The Hague	2 night shelters	90 (44%)
6b					2 youth shelters	13 (72%)
7	2017	October –November	Municipal Auditory Board	Rotterdam	2 night shelters 1 crisis shelter	30 (qualitative case sample)
Total				**7** cities	**16** facilities	**436** participants

*The percentages in the right column relate participants' numbers to the numbers of HS users present at the interview days. No percentage was computed for subsample 4 (an open group without a visitors list) and 7 (a qualitative set of cases)*.

As the results of Table 2 show, about half the interviews (55%) were held in the large cities meaning The Hague, Utrecht, and Rotterdam, with high concentrations of homeless people. The other half (45%) were held in the medium-sized cities of Dordrecht, Eindhoven, Heerlen, and Landgraaf. In most settings, the majority of HS users participated in the study. In one night shelter, the opening hours and the spacious hall hampered blending with HS users. Still, in all facilities the participants represented a saturated sample of the HS users present in the services at the interview days. This was confirmed by the facility staff.

**Figure 2:**
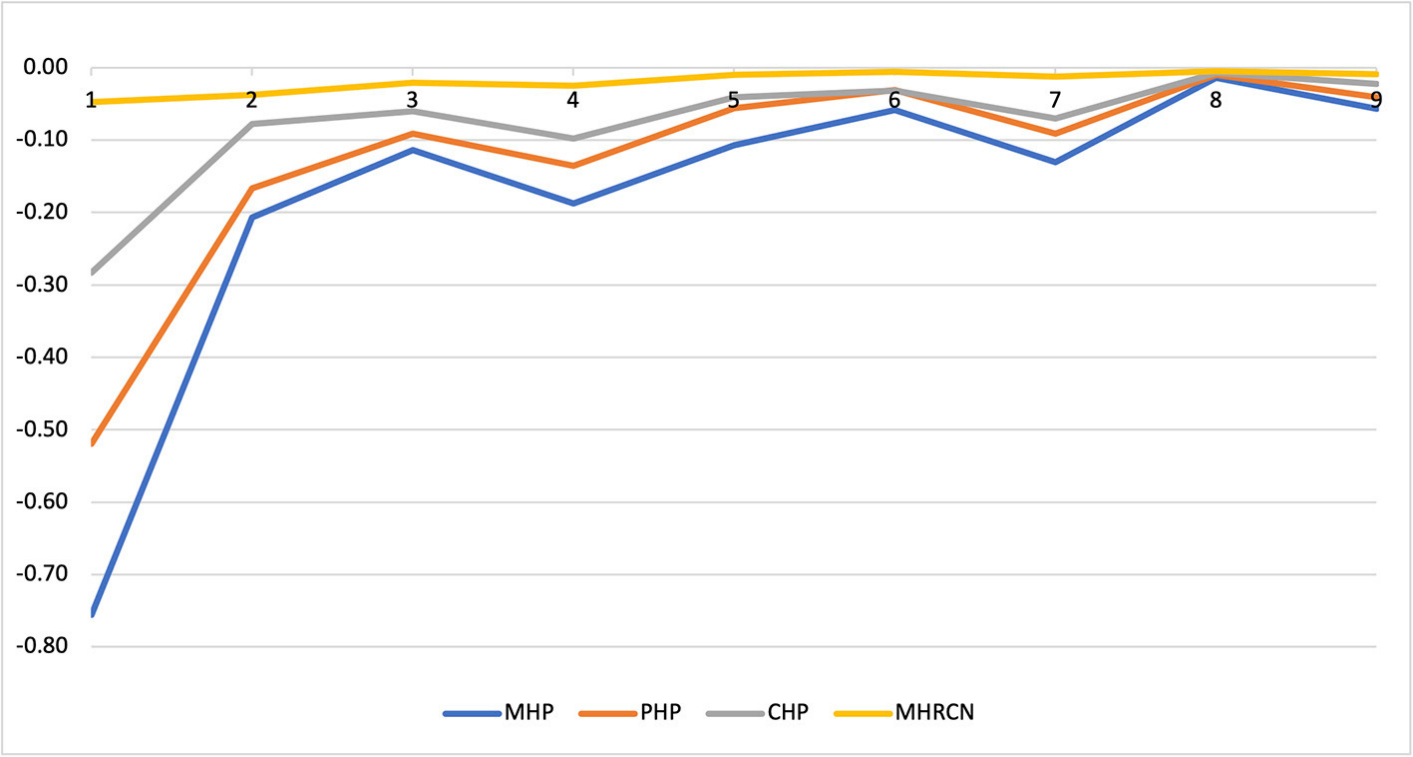
Additional information by subsample.

Recruitment aimed to add settings as long as additional reviews generated new insights in health patterns. Analyses were done for comparing health and needs patterns in the various subsamples (Figure 2). Four clinical integrating assessments were used for providing insight in the diversity of subject recruitment within settings. First, the number of Transdiagnostic Mental Health Features was calculated to assess the burden of Mental Health Problems (MHP) in all subjects. Second, the separate physical health problem scores were used to calculate the number of Physical Health Problems (PHP) in all subjects. Third, the dichotomized codes of Mental Illness, Addiction, Intellectual Impairments, and PHP were used to count the number of Concurrent Health Problems (CHP) in all subjects. Further, the subsample means and Standard Deviations (SD) of MPH, PHP, CHP and Mental Health Related Care Needs (MHRCN) were computed. Finally, an analysis of variance was done based on the subsample means of the subsamples in the left column of Table 2.

Figure 2 presents the results of the analysis. The subsample means of MHP, PHP, CPH,and MHRCN were used to calculate the additional value of successive samples to the cumulative reference. Over the total sample, the mean number MHP was 5.3 (range 0–10, SD 1.9). The mean number of PHP was 1.1 (range 0–7, SD 1.2). The mean sum score of CHP was 2.8 (range 0–4, SD 0.84). The weighted population mean of MHRCN was 1.7 (range 0–2, SD 0.6). The results in Figure 2 show that subsample addition was continued until topic saturation was reached.

Table 3 presents the background characteristics. It shows that most subjects were long-term or intermittently homeless. Chi-square and *t*-tests were run to compare the sample characteristics with reference data on homeless people in the Netherlands. (Supplementary Tables 3, 4 contain the results). The reference data concerns the homeless sample of Netherlands Statistics [CBS; (86, 87)] and the study sample in the evaluation of the Dutch National Homelessness strategy [Coda-G4; (88)].

**Table 3:** Background characteristics (in %).

Sex	Male	81.0
	Female	19.0
Age	18–29 years	19.3
	30–49 years	46.6
	50–64 years	29.6
	65 years or older	4.6
Migration background^a^	Netherlands	47.9
	Other western countries	13.1
	Non-western countries	39.0
	First generation	39.2
	Second generation	12.8
Education: highest level completed	Low	82.3
	Middle	14.9
	High	2.8
Homelessness	Roofless: rough sleepers	7.6
	Roofless: night shelters	67.4
	Houseless: in homeless accomodation	21.1
	Houseless: long term homeless supported living	1.8
	Houseless: independent living with long term support	2.1
	Previous homelessness (ETHOS)	78.8
	Residential instability in past 2 years	91.7

a *According to the CBS definition, someone has a western migration background if he/she or at least one of the parents was born in Europe (excluding Turkey), North America or Oceania. Indonesia and Japan are also considered western countries. Someone who was born, or whose parent(s) was/were born, in any other country is considered as having a non-western migration background*.

No differences were found for sex, but subjects in the HOP-TR sample were significantly older and had less frequent a migration background. Compared to the CBS samples, the educational level of subjects in the HOP-TR sample was lower.

### Assessment Results

#### Health and Needs Patterns

The HOP-TR assessment approach resulted in a rich dataset on health and needs of HS users, containing quantitative and qualitative data in two perspectives. Table 4 presents the result domains and offers a glimpse of the result topics. It shows that the positive health framework was used to structure health data. Additional domains include background characteristics, life history, care history, and a care-needs appraisal. Supplementary Table 1 provides a complete summary of result subjects and topics in the HOP-TR database. Most data are available on the whole sample. As the QoL/QoC questionnaire and the CAN were added since local review 5, this data is only available in a part of the sample (161 subjects).

**Table 4:** HOP-TR assessment results.

**Subjects**	**Main topics**
Physical health	Physical status
	Physical Health Problems
Mental health	Mental status
	Substance use
	Cognition, daily decision making
	Transdiagnostic mental health features
	Mental Illness, Mental Health Problems
	Concurrent Health Problems
Daily functioning	Behavior
	ADL performance & capacities
	Daily activities
	Living situation
Social participation	Social contacts; social support; confidant
	School career; education level; literacy
	Work history; income status
	Police-justice interaction
Quality of Life	Quality of Life
Meaning	Personal goals; personal treatment goals; life orientation
Background	Demographic background; migration; embeddedness
	Highest educational attainments
Life history	Life course
	Previous homelessness, homelessness history; typology;
Care history	Medication; hospital use; care & support use
Care – Needs	Vulnerabilities, Strengths
appraisal	Participant's care appraisal; Quality of Care
	Care Needs Appraisal
	Physical Health Related Care Needs
	Mental Health Related Care Needs
	Future Living Status
	CMH CAPs/scales
	SIX, FR-scale
	CAN

**Figure 3:**
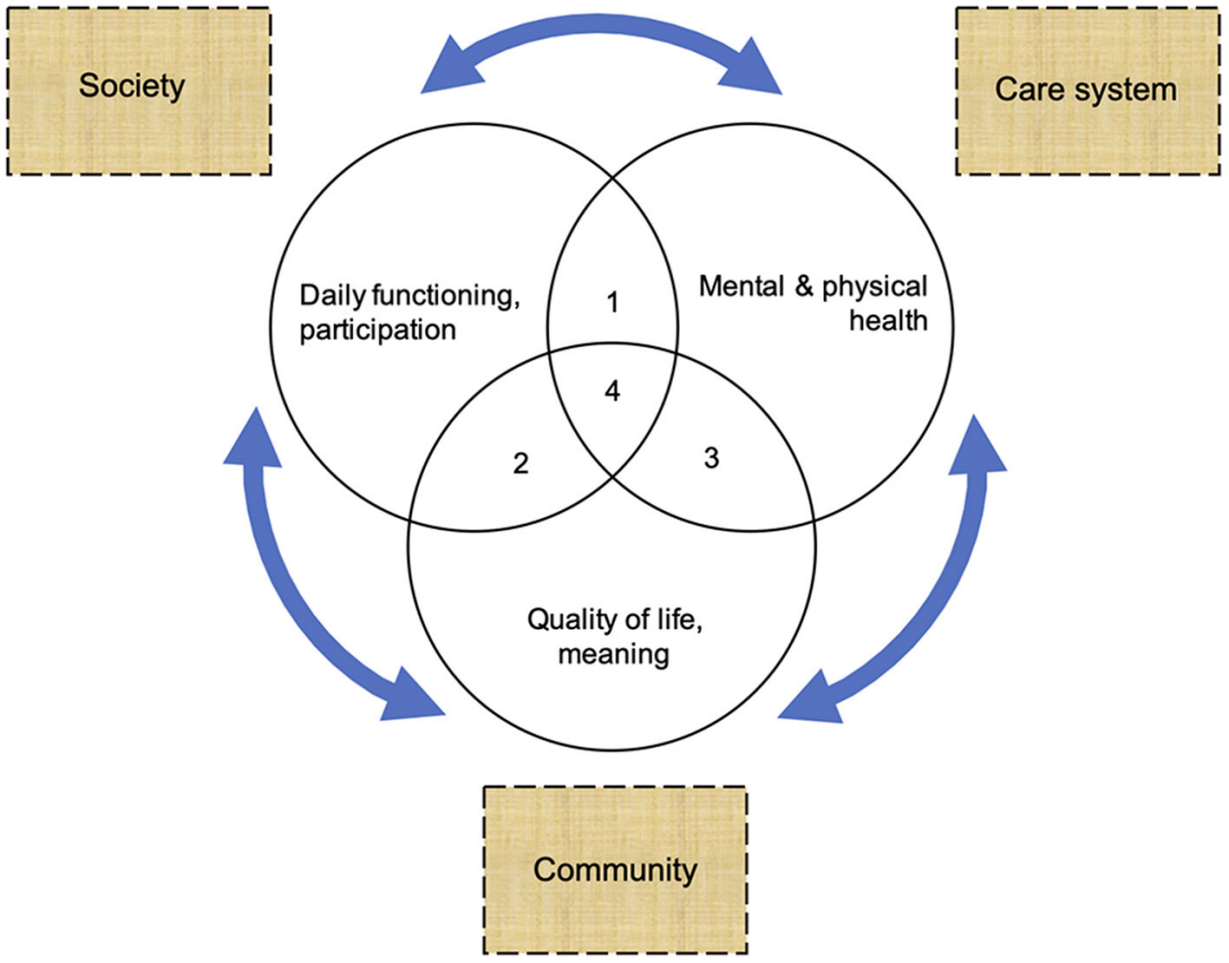
Comprehensive assessment of symptomatic, social, and personal dimensions and conditions to enable recovery.

The three circles in Figure 3 cover the six positive health domains. It clusters health data in symptomatic (mental and physical symptoms), social (daily life and social/societal participation), and personal (quality of life, meaning) health dimensions. It shows how the positive health framework assists in making comprehensive health assessments and translating features into patterns.

These three health dimensions are useful for assessing the whole sample and exploring patterns within the sample. Within the symptomatic dimension, the transdiagnostic mental health results depict and count the burden of mental health problems in the HS users' perspectives. The MHP, PHP, CPH, and MHRCN results, presented in Figure 2, are examples of pattern results reporting on the symptomatic health dimension. The items and sum scores of the SIX and the FR-scale are pattern measures for portraying social and personal health domains. The results on the SIX and on the FR-scale both sum up to a combined score with a maximum of six (51, 52). On the SIX, higher scores relate to better outcomes in terms of social inclusion. By contrast, on the FR-scale higher scores point to higher support needs. The CMH CAPs and scales are pattern codes too, which translate health data into risks and needs assessments regarding symptomatic and social health dimensions.

#### Marginalization – Recovery Patterns (Individual Level)

The next part of the results focuses on drivers in the light of marginalization and recovery (at the level of individual HS users). In a socio-ecological point of view, these are two tail ends of a continuum. The quantitative and qualitative data in Table 4 include information on drivers to both ends of the continuum. Results on care needs and current care, e.g., can be used to identify care gaps. Results on the social network also contain information on the availability of social support. Digit 1 to 4 in Figure 3 will help identify relevant drivers. Therefore, this part of the results identifies care gaps, conditions, or resources relevant to recovery at the level of individual HS users.

#### Marginalization – Recovery Patterns (Network Level)

The third part goes a level deeper. It contextualizes individual marginalization-recovery results in the context of care systems, society, and communities. It aims to explore how mutual responsiveness in treatments, policies, or interactions at the level of networks can be improved. At this level, digit 1 to 4 in Figure 3 will help identify relevant factors at the interfaces between care systems, society, and communities. The results will be used to make a model article on the drivers of recovery.

### Ecosystem Effects

After the first report, the local reviews in Table 2 resulted in a demand for new reports. The regional assessments provided accurate information on health and needs of HS users in two perspectives. From the third report on, assessments of Mental Health Related Care Needs were added, including the population prevalences of SMI. The regional assessments responded well to the information needs of local authorities. Commissioning organizations used the results to evaluate municipal homelessness policies and explore service improvements in dialog with other stakeholders. In each region, the transparent results stimulated reflection and discussion about care provision, network cooperation, care access, and quality of care. In some regions the data forged long-term commitment for care improvement.

The local reviews impacted on HS users as well. At the level of individual HS users, many participants experienced the interview as empowering and hopeful. The process of telling their own stories stimulated reflection on personal goals and alternative plans (89). Likewise, the local reports affected HS users in local and regional networks. The reports gave voice to HS users' perceptions of current care, quality of life, and priorities to shape new perspectives on recovery. Consequently, HS users felt encouraged to actively participate in local stakeholder networks. At the same time, the regional discussions pushed the national debate. The HOP-TR study stimulated a public health dialog at different ecosystem levels. This kept the meta-snowball rolling and resulted in a large sample size.

## Discussion

The HOP-TR study is a recent study among homeless people in the Netherlands. Both in recruitment and assessments, innovative methods were used. This paper describes the recruitment procedure and presents the heart of the assessment approach.

An innovative recruitment strategy was used to collect data in a large sample of HS users. Homeless populations are difficult to access. Homeless people have learned to blend and survive in the streets. Frequently, they are suspicious of authority, which may hamper the decision to participate. The lack of systematic complete and accurate registers of the HS user population impedes probability sampling. Additionally, classic probability sampling in mentally ill homeless populations would overrepresent the less severely ill. Consequently, it is virtually impossible to avoid bias.

In the HOP-TR study, bias was minimized using a naturalistic snowball sampling strategy. Incited by the socio-political context, the meta-snowball at setting level enabled to collect data in various settings and cities. Similarly, snowball sampling of individuals within settings enabled to monitor diversity of subject recruitment until saturation at setting level occurred. A substantial share of the subjects was recruited in night shelters. This might influence the descriptive study results (on separate features) but is irrelevant to the analytical epidemiological results (patterns) about this sample. The results in Figure 2 confirm that recruitment was continued as long as settings generated new insights in health patterns. The recruitment strategy was successful: together, the participants constitute a saturated sample of the adult HS users in the Netherlands in the period 2015–2017.

Comparison of the background characteristics of the HOP-TR sample to Dutch reference samples reveals significant differences. These differences relate to differing recruitment procedures. Fieldwork was executed to collect the HOP-TR data during face-to-face interviews. Therefore, the figures in the HOP-TR sample are population prevalences in a particular part of the Dutch inhabitants. By contrast, the reference data are care prevalences. All subjects in the Coda-G4 reference sample were included in municipal homelessness trajectories (88). The national CBS homelessness figures concentrate on the most visible, nuisance-giving roofless part of the Dutch homeless population. The CBS sample was recruited from administrative data on homeless people included in care programs or municipal homelessness programs (86, 87).

Compared to the CBS sample, the education level in the HOP-TR sample is significantly lower. Again, recruitment differences offer a reasonable explanation. The HOP-TR sample provides a real-life cross-sectional picture based on face-to-face contacts in the homeless services. Various factors might contribute to the lower educational level in the HOP-TR sample, such as a lower literacy, a lower health literacy, and lower skills to get access to support services in the complex Dutch service system. Probably, a part of the HS user population disappears into homelessness again before reaching care access. Overall, is it likely that the lower educational level in the HOP-TR sample is related to different survival strategies.

Further, an innovative assessment approach was developed to enable comprehensive health and needs assessments in a rights-based, recovery-oriented approach. This resulted in a strategy to collect quantitative and qualitative health and needs data in two perspectives. The perspective of the HS users is the primary reference and enriched with professional assessments. The mental health strategy is transdiagnostic. The positive health framework assists to assess health and needs in the symptomatic, social, and personal dimensions of recovery. Specific codes were added to establish needs in an independent, rights-based approach. Referring to Mansell (36), the strategy was developed to enable dialogs and build models to test transdiagnostic interventions in a healthcare ecosystem approach.

Results on symptomatic health dimensions, such as MPH, PHP, CHP, and MHRCN are useful to describe the burden and patterns of health problems in marginalized populations. Recently, the results on patterns of concurrent health problems among Dutch HS users were published (25). In most subjects (95.0%), CHP affected two or more domains (Mental Illness, Addiction, Intellectual Impairments, chronic Physical Health Problems). The results reveal the multi domain character of needs and the relevance of broad, comprehensive assessments.

The main topics of collected health and needs data are summarized in Table 4. Those health and needs results enable to uncover drivers in the pathways to marginalization and recovery. Figure 2 illustrates how contextualization of health and needs results in the positive health framework can assist in meeting the second and third study objective. Local authorities, the primary stakeholders, already used the local reviews to stimulate reflection and discussion about needs, care provision, access, network cooperation, and about quality of care at different geographical ecological levels.

To date, potential applications of the HOP-TR strategy in a healthcare ecosystem approach were only partially used. The regional assessments were centered to descriptive reports on health and needs results. It appears valuable and potentially powerful to use analytical epidemiological HOP-TR results as well in dialogs at different ecosystem levels. Analytical data on marginalized populations such as the homeless are landmarks of quality and functioning of care systems in regions or countries. For example, the SMI/EPA share in homeless populations is a significant quality indicator of the attainment of community care. Additionally, individual and regional assessments can create a solid foundation for ecosystem dialogs concentrating on human rights. The HOP-TR approach produces mirrors which enable to discuss and to shape new perspectives on recovery. The revised health concept is oriented toward individuals (29). It considers health as “the ability to adapt and self-manage, in the face of social, physical and emotional challenges.” The HOP-TR approach is oriented toward societal networks. It results evoke dialog and incite to foster the “network ability to adapt and co-manage in the face of social, physical, cultural, economic, and political challenges.” Therefore, future replications of this protocol might contribute to face societal challenges at different ecosystem levels.

### Limitations

All data were collected in single assessments by a single interviewer. Medical records were not available, except in the protected living setting. Four out of five subjects had not visited any physician within the last 3 months. This shortcoming was cared for using individual assessments by a researcher with an MD professional background.

Single person assessments might induce a bias but offer best health estimates of a hidden part of the Dutch HS population. Further, the data quality is limited to information collected during single encounters. Better care access and additional checkups certainly would provide more reliable descriptive health data.

The HOP-TR database was built over different local reviews, commissioned by municipalities, and service providers. Because the data collection concentrates on HS users in traditional homeless services, population selection bias might limit the generalizability of the results. Evidently, homeless people with insecure or in inadequate housing, such as “sofa surfers” and “work migrants lodging in holiday parks,” are underrepresented. Further, the cross-sectional design overrepresented individuals with complex health problems by assessing the most needing individuals who stay in the facilities the longest time. Considering the hidden nature of this population, the HOP-TR recruitment strategy is the best possible approach to comprehensively assess a representative sample of the Dutch adult HS users in 2015–2017. In the future it can be supplemented with a cohort strategy to better document who uses these services, albeit temporarily.

### Additional Considerations

Finally, we briefly reflect on organizational choices in eventual replications. This study was initiated to obtain health data in a difficult to reach part of the Dutch inhabitants. For pragmatic reasons, all data was collected by a researcher with a professional background as a MD. This methods paper documents where and how the researcher used her background to collect and interpret anamnestic health information and observations, to obtain an overall picture on health and (unmet) health related needs. A medical background is not required for all parts of this approach. All assessments are highly manualized. No specific medical knowledge is necessary to use the open questions, HSup, QoL/QoC questionnaire, the MoCA, or the SCIL. Similarly, the algorithmic summary scales do not require medical knowledge either. Even though the CMH, CAN, and FR-scale were developed for application by health professionals, the instruments are attended with training programs to aid data-collection by interviewers with various backgrounds. By contrast, in difficult to reach underserved populations, clinical knowledge is useful for examining the care history and necessary for assessing the clinical integrating assessments. Therefore, it seems practical to work with trained interviewers with a medical background such as nurses, physicians, or psychologists. Alternatively, a staged approach can be used to organize the data collection and interpretation process. In the NEMESIS study (90, 91), e.g., the basic interviews were executed by interviewers experienced in systematic data collection who were acquaintanced with the instruments after following a several days' course. The presence of one or more psychotic symptoms incited to the execute structured psychiatric reinterviews. Similarly, the HOP-TR assessment strategy can also be organized in a two-stage approach. If for example trained social workers execute the basic interviews, the quality of the entire data collection process could be monitored in a program comparable to that in the NEMESIS study. Finally, as homelessness is a public health issue, the presence of an overall, public health helicopter view within the research team remains essential.

## Data Availability Statement

The raw data supporting the conclusions of this article will be made available by the authors, without undue reservation.

## Ethics Statement

The research ethics committee of the Radboud University Nijmegen Medical Centre approved that our research plan for the scientific use of the management data does not fall within the remit of the Medical Research Involving Human Subjects Act (record number 2018-4463). Therefore, our research plan can be carried out in the Netherlands without an approval by an accredited research ethics committee and without explicit written informed consent of the participants. Consent to participate was obtained. Oral consent in report 1 and 2; in addition to the oral consent also written consent in report 3 to 7.

## Author Contributions

CvE, PBP, KvdV, and PhD participated in the conceptual design of the study. CE drafted the manuscript, collected the data, and performed the analyses. All the authors critically revised the manuscript, contributed to interpretation of the data, and read and approved the final version of the manuscript.

## Conflict of Interest

The authors declare that the research was conducted in the absence of any commercial or financial relationships that could be construed as a potential conflict of interest.

## Footnotes

^1^The Dutch acronym to indicate SMI.

^2^Former minister of health, Els Borst, subsided February 2014 after she was frequently stabbed with a knife. BvU confessed Borst's murder as a divine task, because of her role to bring euthanasia in Dutch legislation (https://www.nrc.nl/nieuws/2016/02/05/bart-van-u-had-goddelijke-opdracht-1588377-a850833). Consequently, a special Committee was appointed to evaluate the quality of juridical operations and decisions. In June 2015, the Committee urgently assigned the Public Prosecutor, police and mental health care sector to improve the follow up of potential safety risks related to (severe) mental illness immediately.
