# Supplementary material for: A Comprehensive Assessment to Enable Recovery of the Homeless: The HOP-TR Study
Source: Front Public Health. 2021 Jul 9;9:661517. doi: 10.3389/fpubh.2021.661517 (PMC8299205; doi:10.3389/fpubh.2021.661517)
Supplement: Supplementary file 1 [file Data_Sheet_1.PDF]

Supplementary table 1. Results of the HOP-TR assessment approach

This table provides a complete summary of the subjects and topics in the HOP-TR assessment approach. It relates the subjects and topics in column II and III to the result domains in column I. Column IV shows the sources of the assessments and refers to the part of this assessment approach. Column V denotes the local reviews of the assessments.

Abbreviations: ASS: Algorithmic Summary Scales; BI: Basic Interviews; CIA: Clinical Integrating Assessments; CAN: Camberwell Assessment of Need; CMH: InterRAI Community Mental Health questionnaire; ETHOS: European Typology of Homelessness and Housing Exclusion; FR: Functional Recovery scale; HSup: Homelessness Supplement; ID: InterRAI intellectual Disability questionnaire; MoCA: Montreal Cognitive Assessment; OQ: Open Questions; Q: Quality; QoC: Quality of Care questionnaire; QoL: Quality of Life questionnaire; SCIL: Screener for Intelligence and Learning Disabilities; SIX: Social Outcomes Index.

S1	Subject	Topics	Source		Sample
Physical health	Self-rated health		CMH	BI	1 – 7
	Physical symptoms	Cardiovascular, Respiratory, Gastrointestinal, Urinary, Neurological, Skin, Fatigue, Emergent conditions	CMH	BI	1 – 7
		Extrapyramidal symptoms	CMH	BI	1 – 7
		Performance of sexual activities for money	CMH	BI	1 – 7
	Pain	Frequency, Intensity, Consistency, Control	CMH	BI	1 – 7
	Physical functioning	Foot problems, Falls, Exercise	CMH	BI	1 – 7
		Standing balance, Stairway performance	HSup	BI	1 – 7
		Potential improvement	CMH	BI	1 – 7
	Continence	Bladder, Bowel	CMH	BI	1 – 7
	Nutritional status	Height, Weight, Nutritional issues	CMH	BI	1 – 7
	Physical disease	Asthma, Diabetes, Hypothyroidism, Migraine, Traumatic brain-injury, Other physical ICD-10	CMH	BI	1 – 7
	Chronic Physical Health Problems	Cardiovascular, Respiratory, Gastrointestinal, Musculoskeletal, Endocrine, Neurological, Malignancy, Underweight/overweight	CIA	CIA	1 – 7
	Physical Health Problems	Number of chronic physical health problems present	CIA	CIA	1 – 7
	Physical disability		CAN	BI	5 – 7
Mental health	Self-rated health	Anxiousness, loss of interest, sadness	CMH	BI	1 – 7
	Mental state indicators	Mood disturbance, Anxiety, Psychosis, Negative symptoms, Sleep problems, Other indicators	CMH	BI	1 – 7
		Psychotic symptoms, Psychological distress, Sleep	CAN	BI	5 – 7
	Substance use	Number of drinks, Drunkenness	CMH	BI	1 – 7
		Alcohol abuse history	HSup	BI	1 – 7
		Time since use of: Inhalants, Hallucinogens, Cocaine, Stimulants, Opiates, Cannabis; Injection drug use	CMH	BI	1 – 7
		Caffeine, Tobacco, Gambling	CMH	BI	1 – 7
		Environmental reactions on substance use	CMH	BI	1 – 7
		Ever diagnosed substance related disorder	CMH	BI	1 – 7
		Alcohol, Drugs/medication	CAN	BI	5 – 7
	Harm to self and others	Indicators of self-injurious behavior	CMH	BI	1 – 7
		Violence ideation, Threatening, Violence to others	CMH	BI	1 – 7
		Extreme behavior disturbance, perpetrator of sexual violence	CMH	BI	1 – 7
		Safety to self, safety to others	CAN	BI	5 – 7

S1	Subject	Topics	Source		Sample
Mental health (continued)	Traumatic life events	Character & time since, Burden, Daily impact, Pattern	CMH	BI	1 – 7
		Traumatic brain injury	CMH	BI	1 – 7
	Behavior	Wandering, Verbal or physical abuse, Social or sexual inappropriate behavior	CMH	BI	1 – 7
	Cognition	Daily decision making, Memory	CMH	BI	1 – 7
		Change in decision making	CMH	BI	1 – 7
		Periodic disordered thinking or awareness	CMH	BI	1 – 7
		Cognitive screening	MoCA	BI	1 – 7
		Intellectual disability screening	SCIL	BI	1 – 7
		Ability to adapt to changes in routine	ID	BI	1 – 7
		Insight degree	CMH	BI	1 – 7
	Competences	Consent treatment, Clinical records, Manage property	CMH	BI	1 – 7
	Psychiatric diagnoses	DSM-5 group & text diagnoses, Intellectual disability	CIA	CIA	1 – 7
	Transdiagnostic mental health features	Trauma, Depression, Psychosis, Agitation, Identity, Neurocognitive impairments, Intellectual impairments, Gender, Somatization	CIA	CIA	1 – 7
	Mental Health Problems	Number of transdiagnostic mental health features present	CIA	CIA	1 – 7
	Mental illness	Any transdiagnostic mental health features, not being addiction or intellectual impairments	CIA	CIA	1 – 7
	Concurrent Health Problems	Sum score of the dichotomized results of 4 variables: Mental Illness, Addiction, Intellectual Impairments, chronic Physical Health Problems	CIA	CIA	1 – 7
Daily functioning	Activities of daily living (ADL)	Personal hygiene, Locomotion, Toilet use, Toilet transfer, Eating performance	CMH	BI	1 – 7
		Telephone, Transport	CAN	BI	5 – 7
	Instrumental ADL	Meal preparation, Housework, Finance, Medications, Phone use, Shopping, Transportation	CMH	BI	1 – 7
		Food, Household skills, Self-care	CAN	BI	5 – 7
	Communication abilities	Making self-understood, Understanding, Hearing, Seeing	CMH	BI	1 – 7
		Literacy: ability to handle written information in Dutch	SCIL	BI	1 – 7
		Basic education	CAN	BI	5 – 7
	Living conditions	Previous living conditions	HSup	BI	1 – 7
		Living arrangement, Housing instability past 2 yr.	CMH	BI	1 – 7
		Accomodation, family	SIX	ASS	1 – 7
		Daily living and self-care	FR	BI	1 – 7
		Accomodation	CAN	BI	5 – 7
Social participation	Contact with important others	Partner, Children, Friends	HSup	BI	1 – 7
		Friends	SIX	ASS	1 – 7
		Social contacts	FR	BI	1 – 7
		Confidant, Strong relationship with family	CMH	BI	1 – 7
		Company of others, Intimate relations, Sexual expression, Child care	CAN	BI	5 – 7
	Social participation	Social activities, Social interaction, Community involvement	CMH	BI	1 – 7
		Number of days went out, time alone	CMH	BI	1 – 7
	Unsettled relationships	Conflict or repeated criticism	CMH	BI	1 – 7

S1	Subject	Topics	Source		Sample
Social participation (continued)	Informal support	Presence & relation to 2 informal helpers, Contact focus, Contact frequency	CMH	BI	1 – 7
		Stress or dysfunction	CMH	BI	1 – 7
	Work or purposeful daily activities	Ever fulfilled regular job, Work for >= 1 year, Kind and periods of working activities	HSup	BI	1 – 7
		Work, study or purposeful daily activities	FR	BI	1 – 7
		Work status	HSup	BI	1 – 7
		Employment	SIX	ASS	1 – 7
		Occupation, Paid work	CAN	BI	5 – 7
		Unemployment risk indicators, Persistent unemployment (past 2 years)	CMH	BI	1 – 7
		Reason job loss	HSup	BI	1 – 7
Q	Perceived Quality of Life	Life, Living situation, Social relations, Physical health, Mental health	QoL	BI	5 – 7
Meaning	Life goals	Personal goals	OQ	BI	1 – 7
		Personal treatment goals, Plans for future needs	CMH	BI	1 – 7
	Life orientation	Positive outlook	CMH	BI	1 – 7
		Meaning & recovery	CAN	BI	5 – 7
Background	Birthdate; sex		CMH	BI	1 – 7
	Family	Partner, Children, Age of children, Civil status	HSup	BI	1 – 7
	Migration background	Country of birth, Parents' countries of birth Migration history, Asylum status, Bond with region	HSup	BI	1 – 7
	Education	Highest educational attainments	HSup	BI	1 – 7
Life history	Life course		HSup	BI	1 – 7
	Homelessness	Typology	ETHOS	BI	1 – 7
	Previous homelessness	According to ETHOS	ETHOS	BI	1 – 7
	Current homelessness	Date, Origin, Immediate cause of current period	HSup	BI	1 – 7
	Overnight stay	Overnight stay prior to current stay, Start shelter use (this period)	HSup	BI	1 – 7
Care history	Current care	Opening date, Payment source, Insurance	HSup	BI	1 – 7
		Trajectory type, Case management, Current medical/mental/social care	HSup	BI	1 – 7
		Disciplines, Treatment modalities, Focus of intervention	CMH HSup	BI	1 – 7
	Medication use	List of all medications	CMH	BI	1 – 7
		Adherence, Intentional misuse, Drug allergy	CMH	BI	1 – 7
		Side effects medication	CAN	BI	5 – 7
	Non-psychiatric treatment	Overnight hospital stays, Emergency room visits, Physician visits	CMH	BI	1 – 7
	Mental health service use	Last community mental health contact	CMH	BI	1 – 7
		Psychiatric admissions: Time since, Number, Lifetime, Age at first, Involuntary	CMH	BI	1 – 7
		Current care: disciplines, focus of intervention	CMH	BI	1 – 7
	Financial situation	Income source, Financial status, Outstanding debts	HSup	BI	1 – 7
		Trade-offs	CMH	BI	1 – 7
		Money, welfare benefits	CAN	BI	5 – 7
	Work support	Work orientation, Work arrangement, Education program	CMH	BI	1 – 7

S1	Subject	Topics	Source		Sample
Care hist.	Police intervention	Violent behavior, Non-violent behavior	CMH	BI	1 – 7
	Incarceration status	Time since latest incarceration	CMH	BI	1 – 7
	Justice interaction	Probation/parole, Court diversion program, Restraining orders, Community treatment order	CMH	BI	1 – 7
		Juridical problems	CAN	BI	5 – 7
Care appraisal	Participant's care appraisal	Appraisal of current care, Casemanager known	HSup	BI	1 – 7
		Care resistance	CMH	BI	1 – 7
		Quality of care, Confidence in casemanager	QoC	BI	5 – 8
		Information about condition and treatment	CAN	BI	5 – 7
	Conjoint care-needs appraisal	Vulnerabilities, Strengths	CIA	CIA	1 – 7
		Physical Health-Related Needs	CIA	CIA	1 – 7
		Mental Health-Related Care Needs	CIA	CIA	1 – 7
		Social Domain Needs	CIA	CIA	1 – 7
	Professional care-needs appraisal (continued)	Future Living Status; Physical Health-Related Needs to Housing	CIA	CIA	1 – 7
		Care versus needs	CIA	CIA	1 – 7
		Care appraisal at moment social decline, Care appraisal of current care	CIA	CIA	1 – 7
		Traject responsibility best	CIA	CIA	1 – 7
	InterRAI CAPs & scales	Positive symptoms, Depression, Mania, Traumatic life-events, Sleep disturbance, Cognitive performance, Substance use, Smoking, Exercise, Aggressive behavior, Harm to others, Self-harm, Self-care, Interpersonal conflicts, Informal support, Social relationships, Criminal activities, Personal finance, Education & employment, Weight management, ADL	CMH	ASS	1 – 7
	Camberwell Assessment of Need	Accomodation, Food, Household skills, Self-care, Occupation, Physical health, Psychotic symptoms, Information about condition and treatment, Psychological distress, Safety to self, Safety to others, Alcohol, Drugs, Company to others, Intimate relationships, Sexual expression, Child care, Basis education, Telephone, Transport, Money, Welfare benefits, Paid work, Side-effects medication, Recovery, Legal case, Sleep, Caregiver information, Caregiver distress	CAN	BI	5 – 7
	Social Outcomes Index	SIX employment, SIX accomodation, SIX family, SIX friends	SIX	ASS	1 – 7
	Functional Recovery scale	FR daily living & self-care, FR work study household, FR social contacts	FR	BI	1 – 7
