# Supplementary material for: A Comprehensive Assessment to Enable Recovery of the Homeless: The HOP-TR Study
Source: Front Public Health. 2021 Jul 9;9:661517. doi: 10.3389/fpubh.2021.661517 (PMC8299205; doi:10.3389/fpubh.2021.661517)
Supplement: Supplementary file 2 [file Data_Sheet_2.PDF]

Supplementary table 2. Codes Homelessness Supplement and Clinical Integrating Assessments

This table provides a complete description all Homelessness Supplement (HSup) codes and Clinical Integrating Assessments (CIA) in the HOP-TR assessment approach. It relates the codes in column II to the result domains in column I. Column III shows the operationalization. The characters in column IV indicate if the output belongs to the HSup or to the CIA.
Some specific CMH and ID codes were added, if they were specifically used in the concerned CIA operationalization.

Abbreviations: CMH: InterRAI Community Mental Health questionnaire; Daily f.: Daily functioning; ETHOS: European Typology of Homelessness and Housing Exclusion; ID: InterRAI intellectual Disability questionnaire; MoCA: Montreal Cognitive Assessment; Q: Quality; SCIL: Screener for Intelligence and Learning Disabilities; TDF: transdiagnostic mental health features.

S2	Code	Operationalization	Output
Physical health	Chronic cardiovascular disease	0: No; 1: Hypertension, hypercholesterolemia;<br>2: Ischemic heart disease, heart failure and/or severe intermittent claudication/aortic bifurcation prosthesis;<br>3: Intermittent claudication	CIA
	Chronic infectious disease	0: No; 1: Viral hepatitis and/or HIV	CIA
	Chronic gastrointestinal problems	0: No; 1: Esophagitis or gastritis; 2: Liver cirrhosis;<br>3: Chronic pancreatitis or m. Crohn	CIA
	Chronic musculoskeletal problems	0: No; 1: Amputation leg or arm; 2: Other	CIA
	Chronic neurological disease	0: No; 1: Stroke; 2: Polyneuropathy 3: Parkinson disease or other chronic neurological diseases	CIA
	Chronic respiratory disease	0: No asthma or COPD; 1: Asthma or COPD present	CIA
	Chronic thyroid disease	0: Absent; 1: Hypothyroidism or hyperthyroidism present	CIA
	Diabetes mellitus	0: No; 1: Yes	CMH
	Malignancy	0: No; 1: Malignancy in history - cured;<br>2: Malignancy - need of active follow up or treatment	CIA
	Underweight/overweight	0: Normal weight (BMI >=18.5 & BMI<25)<br>1: Underweight (BMI<18.5);<br>2: Obesity (BMI 25-30);<br>3: Severe obesity (BMI>=30)	CMH
	Visual impairments	0: No; 1: Any difficulty to see in normal light	CIA
	Auditory impairments	0: No; 1: Any difficulty to hear	CMH
	Physical Health Problems	Sum score: number of Physical Health Problems listed above<br>0: No chronic Physical Health Problems known;<br>1: One or more Physical Health Problems present	CIA
Mental health	TDF addiction	0: Absent; 1: Previous use; 2: Current use	CIA
	TDF trauma	0: Absent; 1: Present in relation to prior trauma;<br>2: Present with concern for immediate safety	CIA
	TDF depression	0: Absent; 1: Present	CIA
	TDF psychosis	0: Absent; 1: Present	CIA
	TDF agitation	0: Absent; 1: Present	CIA
	TDF neurocognitive impairment	0: Absent; 1: Present	CIA
	TDF intellectual impairments	0: Absent; 1: Present	CIA
	TDF identity	0: Absent; 1: Present	CIA
	TDF gender	0: Absent; 1: Present	CIA
	TDF somatization	0: Absent; 1: Present	CIA
	TDF personality	0: Absent; 1: Maybe; 2: Paranoid; 3: Schizoid; 4: Schizotypal;<br>5: Antisocial; 6: Borderline; 7: Histrionic; 8: Narcissistic;<br>9: Avoidant; 10: Dependent; 11: Obsessive-compulsive;<br>12: Personality change due to another medical condition	CIA

S2	Code	Operationalization	Output
**Mental health (continued)**	Mental Health Problems	Sum score: number of TDF present (Addiction, Agitation, Anxiety, Depression, Intellectual impairments, Neurocognitive impairments, Problematic Personality, Psychosis, Somatization, Trauma)	CIA
		0: No Mental Health Problems known; 1: One or more Mental Health Problems present	
	Mental illness	One or more TDF (Agitation, Anxiety, Depression, Neurocognitive impairments, Problematic Personality, Psychosis, Somatization, Trauma), excluding Addiction and Intellectual impairments	CIA
	Concurrent Health Problems	Sum of the dichotomous scores of (Mental Illness, Addiction, Intellectual Impairments, Physical Health Problems)	CIA
	History of alcohol abuse:	0: Absent; 1: Present	HSup
	Present alcohol abuse:	0: Absent; 1: Present	HSup
	Age of first drugs use	Age (years)	HSup
	Ability to adapt to changes in routine	0: Adjusts easily; 1: Some troubles; 2: A lot of troubles to small changes in routine	ID
	Intellectual disability screening	0: Probability of (mild, moderate) intellectual disabilities absent; 1: Probability present	SCIL
	Cognitive screening	0: Normal (>25) 1: Abnormal (<26), pointing at mild, moderate or severe cognitive impairments	MoCA
**Daily f.**	Stand balance performance	0: Able without any effort; 1: Able with effort; 2: Unable	HSup
	Stairway performance	0: Able without any effort; 1: Able with effort; 2: Unable	HSup
	Ability to handle written information in Dutch	0: Low literacy is probably absent; 1: Low literacy is probably present	HSup
**Social and societal participation**	Any contact with partner/children/friends?	0: No; 1: Yes	HSup
	Bond with region	0: No bond with region; 1: Born and bred; 2: Work; 3: Family or friend; 4: Other bond to region	HSup
	Work experience	Kinds and periods of working activities? String variable	HSup
	Work experience	Ever fulfilled regular job in Dutch society? 0: No; 1: Yes 0: no; 1: yes	HSup
	Work status	0: Never employed; 1: Persistent unemployed; 2: Currently unemployed; 3: Employed with benefit; 4: Employed; 5: Retired; 6: Student	HSup
	Work duration	One year or more regular or protected work in the Netherlands? 0: No; 1: Yes	HSup
	Current work	0: No job; 1: Job, retaining unemployment benefits; 2: Paid job	HSup
	Reason job loss	String variable	HSup
	Income status	0: No income; 1: Job payment; 2: Benefit or grant; 8: Other	HSup
	Financial status	0: No debts; 1: <2000 € 2: 2000-5000 €; 3: 5000-10000 €; 4: 10000-50000 €; 5: > 50000 €; 8: Amount unknown	HSup
**Q.**	Personal quotes	String variable	HSup
	Life goals	String variable	HSup
	Personal treatment goals	String variable	CMH

S2	Code	Operationalization	Output
Background characteristics	Partner	0: No; 1: Yes	HSup
	Children	0: No;<br>1: Yes; if yes, then number of children? Of how many partners?	HSup
	Age of children	0: No children; 1: Minors <18 years old;<br>2: Only grown-up children in adult age	HSup
	Civil status	1: Never married; 2: Married; 3: Partner or significant other;<br>4: Widowed; 5: Separated; 6: Divorced	CMH
	Migration background: country of birth	0: Native; 1: Foreign - Western European countries (North America, Oceania, Indonesia, Japan, Europe except Turkey);<br>2: Foreign - other countries	HSup
	Migration generation: country of birth (participant & parents)	0: Native; 1: Foreign - first generation;<br>2: Foreign - second generation	HSup
	Asylum status	0: No; 1: Temporary; 2: Permanent	HSup
	Education: highest attainments	1: No education; 2: Special education; 3: Primary school;<br>4: Vocational education - lowest; 5: Secondary school - lowest;<br>6: Vocational education - middle; 7: Secondary school - middle;<br>8: Vocational school - highest; 9: Secondary school - highest;<br>10: University	HSup
Life history	Life course	String variable	HSup
	Previous living accomodation	1: Housing in ownership; 2: Rented - housing association;<br>3: Private rented; 4: Camping site; 5: Living with family or friends; 8: Other or inapplicable	HSup
	Homeless date	Date (month, year)	HSup
	Homelessness type	0: Precariously housed; 1: Homeless without program;<br>2: Homeless in program;<br>3: Asylum seeker without residence permit; 8: Stable housed	HSup
	Homeless origin	String variable	HSup
	Immediate cause of current homelessness	0: Not homeless; 1: Financial problems; 2: Relational conflict with partner or parents; 3: Offence of the opium act;<br>4: Incarceration; 5: Immigration/residence abroad;<br>6: Giving nuisance; 7: Other	HSup
	Overnight stay prior to current accomodation	1: Independent living; 2: Living with family permanently;<br>3: Living with family temporarily; 4: Living unregistered;<br>5: Institution; 6: Crisis shelter; 7: Incarceration; 8: Night shelter;<br>9: Sleeping rough; 10: Other	HSup
	Previously homeless	0: No; 1: Yes	HSup
Care history	Income source	0: No income;<br>1: Job payment; 2: Employee compensation;<br>3: Unemployment benefit; 4: Disability benefit;<br>5: Sickness benefit; 6: Retirement pension;<br>7: Student grants; 8: Other	HSup
	Medical insurance?	0: No; 1: Yes	HSup
	Outstanding debts	0: No debts; 1: Debts - not in track; 2: Debts - instalment plan;<br>3: Debt restructuring	HSup
	Current care: opening date	Date	CMH
	Current care: stadium	0: Not in care; 1: Start up; 2: Settled care	HSup
	Current care: payment source	1: Public longterm care insurance (WLZ)<br>2: Public health insurance (Zvw); \<br>3: Judicial; 4: Municipal social services;<br>8: Responsibility unknown or payment not yet available	HSup

S2	Code	Operationalization	Output
Care history (continued)	Current care: social domain service arrangement?	0: No; 1: Submitted; 2: Granted	HSup
	Current care: trajectory type	0: No trajectory; 1: Living-working trajectory;<br>2: Living-working with additional support;<br>3: Probation and after-care services;<br>4: Integrated mental care (ambulant); 5: Care facility;<br>6: Hospital or long term care facility	HSup
	Current care: case management	0: No case management; 1: Municipal inquiries office;<br>2: Social domain; 3: Mental care (including psychiatric department or hospital); 4: Probation and after-care services;<br>5: Youth care; 6: hospital care (non-psychiatric);<br>7: Primary care (GP, nurse practitioner); 8: Other	HSup
	Current social care	0: Not in care; 1: Referred or waiting list; 2: In care	HSup
	Current medical care	0: Not in care; 1: Referred or waiting list; 2: In care	HSup
	Current mental care	0: Not in care; 1: Referred or waiting list;<br>2: Outreach or regular care	HSup
	Receives integrated multidisciplinary mental care	0: No; 1: In guidance to; 2: Receives	HSup
	Mental health - unidisciplinary	0: No; 1: Yes; if yes, then name/discipline/goal or focus	HSup
	Mental health - multidisciplinary	0: No; 1: Yes; if yes, then name/discipline/goal or focus	HSup
	Intellectual impairments - unidisciplinary	0: No; 1: Yes; if yes, then name/discipline/goal or focus	HSup
	Intellectual impairments - multidisciplinary	0: No; 1: Yes; if yes, then name/discipline/goal or focus	HSup
	Addiction - multidisciplinary	0: No; 1: Yes; if yes, then name/discipline/goal or focus	HSup
	General practitioner	0: No; 1: Yes; if yes, then name/discipline/goal or focus	HSup
	Medical specialist(s)	0: No; 1: Yes; if yes, then name/discipline/goal or focus	HSup
	Social worker (single, no team)	0: No; 1: Yes; if yes, then name/discipline/goal or focus	HSup
	Social neighborhood team	0: No; 1: Yes; if yes, then name/discipline/goal or focus	HSup
	Social & legal support services	0: No; 1: Yes; if yes, then name/discipline/goal or focus	HSup
	Housing support services	0: No; 1: Yes; if yes, then name/discipline/goal or focus	HSup
	Language support services	0: No; 1: Yes; if yes, then name/discipline/goal or focus	HSup
	Job-coach	0: No; 1: Yes; if yes, then name/discipline/goal or focus	HSup
	Youth services	0: No; 1: Yes; if yes, then name/discipline/goal or focus	HSup
	Youth shelter	0: No; 1: Yes; if yes, then name/discipline/goal or focus	HSup
	Day shelter/day care	0: No; 1: Yes; if yes, then name/discipline/goal or focus	HSup
	Daily living support	0: No; 1: Yes; if yes, then name/discipline/goal or focus	HSup
	Family support	0: No; 1: Yes; if yes, then name/discipline/goal or focus	HSup
	Lawyer	0: No; 1: Yes; if yes, then name/discipline/goal or focus	HSup
	Probation and after-care services	0: No; 1: Yes; if yes, then name/discipline/goal or focus	HSup
Care appraisal	Personal appraisal current care	String	OQ
	Case manager known?	0: No; 1: Yes	HSup
	Case manager confidence?	0: No; 1: Yes	HSup
	Vulnerabilities	String	OQ; CIA
	Strengths	String	
	Physical Health-Related Needs	String	CIA
	Physical Health-Related Needs regarding to Housing	0: No specific physical health related needs;<br>1: Compensation impairments regarding staircase or wet cell;<br>2: Physical health related need of circadian supervision and/or multidisciplinary treatment	CIA

<table>
<tr><th>S2</th><th>Code</th><th>Operationalization</th><th>Output</th></tr>
<tr><td rowspan="6">Care appraisal (continued)</td><td>Mental Health-Related Care Needs</td><td>0: No MH related care needs;
1: Treatment needs because of mental health problems;
2: Treatment needs because of severe mental illness</td><td>CIA</td></tr>
<tr><td>Future Living Status</td><td>0: Uncertain; 1: Independent living; 2: Assisted living in social domain; 3: Residence for people with mental, intellectual or physical disabilities; 4: Long-term care facility</td><td>CIA</td></tr>
<tr><td>Care versus Needs</td><td>String</td><td>CIA</td></tr>
<tr><td>Care-Needs Appraisal at moment of social decline</td><td rowspan="2">0: No decay; 1: Decay but NO NEED; 2: Decay MET NEED; 3: Decay UNMET NEED; 4: UNMET NEED and blacklisted; 5: UNMET NEED system failure; 6: UNMET NEED run away shrieking; 8: Overmet need; 9: Difficult to evaluate, uncertain</td><td>CIA</td></tr>
<tr><td>Care-Needs Appraisal of current care</td><td>CIA</td></tr>
<tr><td>Traject Responsibility Best</td><td>1: Outreach team; 2: Mental care;
3: Care for people with intellectual disabilities;
4: Social domain - assisted living and/or daytime activities;
5: Social domain - living and/or supported work;
6: Court diversion/support program</td><td>CIA</td></tr>
</table>
