# Supplementary material for: A Comprehensive Assessment to Enable Recovery of the Homeless: The HOP-TR Study
Source: Front Public Health. 2021 Jul 9;9:661517. doi: 10.3389/fpubh.2021.661517 (PMC8299205; doi:10.3389/fpubh.2021.661517)
Supplement: Supplementary file 3 [file Data_Sheet_3.PDF]

Supplementary table 3. Comparison of the HOP-TR and CODA-G4 samples

	HOP-TR		CODA-G4		
**Total sample**	n = 436		n = 513		
Mean age (years)	45.0		40.3		$t = 7.69$, $p < 0.01$
Standard deviation	13.09		13.29		
Age range	18-75		18-71		
Male	353	81.0%	391	76.2%	χ^2 (1) = 3.13, p 0.08
Female	83	19.0%	122	23.8%	
Native	209	47.9%	202	39.4%	χ^2 (1) = 7.03, p 0.01
Migration background	227	52.1%	311	60.6%	
**Adults**	n = 398		n = 410		
Mean age (years)	45.0		40.3		$t = 5.74$, $p < 0.01$
Standard deviation	11.55		11.72		
Age range	23-75		23-71		
Male	329	82.7%	329	80.2%	χ^2 (1) = 0.81, p 0.37
Female	69	17.3%	81	19.8%	
Native	192	48.2%	164	40.0%	χ^2 (1) = 5.57, p 0.02
Migration background	206	51.8%	246	60.0%	
**Young adults**	n = 38		n = 103		
Mean age (years)	20.1		20.2		t = 0.41, p 0.68
Standard deviation	1.29		1.31		
Age range	18-22		18-22		
Male	24	63.2%	62	60.2%	χ^2 (1) = 0.10, p 0.75
Female	14	36.8%	41	39.8%	
Native	17	44.7%	38	36.9%	χ^2 (1) = 0.72, p 0.40
Migration background	21	55.3%	65	63.1%	

Supplementary table 3 compares background characteristics of the HOP-TR sample with the CODA-G4 sample (http://www.codag4.nl/Coda_G4_rapporten,78; table 2.1, p. 17). The CODA-G4 study is a multisite cohort study for monitoring the Dutch national action plan to end homelessness, which ran from 2011-2013. All subjects were included in municipal homelessness trajectories.
The CODA-G4 report describes the background characteristics of young adults (18-22 years) and adults (23 years and older) separately. The numbers of the subsamples were used to calculate mean age, gender, and migration background of the total CODA-G4 sample, presented in the upper section of supplementary table 1. The standard deviations of the age distributions in the CODA-G4 sample were unknown but calculated under the assumption of equal distributions.

Comparison of the HOP-TR sample with the CODA-G4 sample reveals that subjects in the HOP-TR sample are older. Also, the share of subjects with a migration background is significantly lower. Observed differences in gender can be explained by chance.

Supplementary table 4. Comparison of the HOP-TR sample with CBS samples

	**HOP-TR** 2015-2017		**CBS** 2015-2017		
Male	353	81.0%	26333	82.3%	χ^2 (1) = 0.52, p.47
Female	83	19.0%	5667	17.7%	
Age 18 - 29	84	19.3%	10500	33.0%	χ^2 (6) =15262 p <0.01
Age 30 - 49	203	46.6%	15867	49.3%	
Age 50 - 65	129	29.6%	5600	17.3%	
Age 65 and older	20	4.6%	0		
Native	209	47.9%	14300	44.7%	χ^2 (1) = 14105, p <0.01
Migration background	227	52.1%	17700	55.3%	
Education low	359	82.3%	20143	65.5%	χ^2 (4) = 54.7, p <0.01
Education middle or high	65	14.9%	9378	30.5%	
Education high	12	2.8%	1230	4.0%	

Supplementary table 4 compares background characteristics of the HOP-TR sample with the yearly samples underlying the Dutch homelessness estimates of Netherlands Statistics (CBS). The CBS homelessness figures concentrate on the most visible, nuisance-giving roofless part of the Dutch homeless population. The CBS sample was recruited from administrative data on homeless people included in care programs or municipal homelessness programs.
In 2015-2017, the number of homeless people in the Netherlands fluctuated from 31000 (2015), and 30500 (2016), to 34500 (2017). The results in the right column reflect the means of the corresponding numbers in 2015-2017.

Supplementary table 4 shows that gender in both samples is the same. In comparison to the CBS samples, subjects in the HOP-TR sample are older, more often native, and low-educated. All differences are highly significant.
